# Dominant physical mechanisms governing the forced-convective cooling process of white mushrooms (*Agaricus bisporus*)

**DOI:** 10.1007/s13197-020-04402-9

**Published:** 2020-04-15

**Authors:** Razieh Salamat, Hamid Reza Ghassemzadeh, Seyed Faramarz Ranjbar, Jochen Mellmann, Hossein Behfar

**Affiliations:** 1 Department of Biosystem, University of Tabriz, Tabriz, Iran; 2 Department of Postharvest Technology, Leibniz Institute for Agricultural Engineering and Bioeconomy (ATB), Potsdam, Germany; 3 Department of Mechanical Engineering, University of Tabriz, Tabriz, Iran

**Keywords:** Mushrooms, Forced-air cooling, Mathematical modelling, Computational fluid dynamics (CFD)

## Abstract

Nowadays, numerical modelling has been extensively converted to a powerful instrument in most agricultural engineering applications. In this study, a mathematical model was developed to simulate the forced-air cooling process of mushroom. The simulation was performed in CFD code Fluent 19.2 and the conservative mass, momentum and energy equations were solved within the package. The accuracy of the model was then quantitatively validated against experimental data and very good agreement was achieved ($Root{-}Mean{-}Square Error\, \left( {RMSE} \right) \simeq 3.8 \%$). It was confirmed that in addition to convective mode, water evaporation makes a major contribution in mushroom cooling. According to the results, the developed model was able to predict the velocity and temperature profiles with a reasonable accuracy. It also has a potential to be used in design and optimization of such processes.

## Introduction

Mushroom production has substantially been increased in last decades, since it has an important role in human diet due to its unique flavor as well as its high nutritional value (Tao et al. 2006; Lagnika et al. 2012). Postharvest handling of mushroom, however, is very challenging because of its short shelf life of three to four days at ambient temperature (Tao et al. 2006; Burton et al. 1987). High respiration rate (more than 60 mg CO_2 _kg^−1^ h^−1^) classifies mushroom among high perishable commodities (Xiangyou et al. 2014). Moreover, due to its porous structure, it is largely subjected to water loss, which will considerably reduce its marketability because of both quality and weight loss.

Temperature management has long been proven to be the most important factor to ensure the postharvest quality and extend shelf life of fruits and vegetables (Nalbandi et al. 2016; Delele et al. 2013a, b). Precooling, that is, removal the field heat from the products as soon as possible after harvesting, in particular, is a critical step in the postharvest cold chain (Defreaye et al. 2013; Dehghannya et al. 2010) since it significantly slows down the respiration rate and therefore prevents the temperature increasing in product surrounding (Thompson et al. 2001). The respiration rate of mushroom at 20 °C is almost 5 times higher than that of 0 °C (Iqbal et al. 2009). High respiration rates not only increase water loss (Zhao et al. 2016) and tissue aging (Tao et al. 2006), but also reduce the nutritional quality of products when they are consumed during the respiration (Zhao et al. 2016; Aswaney 2007). According to Thompson et al. (2001), a considerable amount of decay, shriveling and the loss of fresh, glossy appearance are observed as a result of just four-hour delay in mushroom precooling.

Room cooling, forced-air cooling, vacuum cooling and hydro-cooling are the cooling techniques applied for agricultural products (Kader 2002). Vacuum cooling is amongst others a rapid cooling technique commercially used for uniform cooling of mushroom. Nevertheless, its high capital and operating costs are regarded as a limiting factor (Kader 2002) when it comes into play especially in developing countries. In addition, considerable weight losses are incurred during vacuum cooling due to the nature of the process (Burton et al. 1987).

Forced-air cooling is known as the most common applied technique (Pathare et al. 2012; Dehghannya et al. 2011) which is almost adapted for all types of agricultural products (Kader 2002). However, several parameters affect forced-air cooling performance with regard to cooling uniformity, cooling rate and energy consumption within the system. For example, cooling uniformity depends to large extend on packages design (the position, area and shape of vents) and their arrangement (Nalbandi et al. 2016; Defraeye et al. 2013; Delele et al. 2013a, b; Ferrua and Singh 2009a; Dehghannya et al. 2011; Wu et al. 2018). This is because the products are cooled by forcing cooled air through the packages (Pathare et al. 2012; Aswaney 2007) during the forced-air cooling process. In addition, the cooling rate is adversely affected by poor airflow distribution inside the package (Ferrua and Singh 2009c; Dehghannya et al. 2011). The cooling air characteristics (temperature, velocity and relative humidity) also have a considerable influence on cooling rate. The first critical step to study how these factors affect the process is gaining a comprehensive understanding of physical mechanisms occurring during forced convection cooling and their interactions. Biological products are complicated in physical structure and have a wide variability which make both experimental and model-based investigations challenging (Pathare et al. 2012). Despite this fact, the airflow behavior and temperature distribution inside the horticultural packages have been widely discussed, both, experimentally (Wu et al. 2018; Bideau et al. 2018) and numerically (Zou et al. 2006a, b; Ferrua and Singh 2009a,b; Dehghannya et al. 2011; Defraeye et al. 2013,2014; Delele et al. 2013a, b; Nalbandi et al. 2016).

In spite of representing more realistic results, experimental studies are always time-consuming, more expensive and situation-specific as well (Zou et al. 2006a; Han et al. 2015). Due to the advances in computational resources and today’s computers, modelling and simulation have been converted to a pivotal tool in all engineering applications such as postharvest engineering. In recent years, computational fluid dynamics (CFD), particularly, has been of increasing popularity due to obtaining airflow pattern and temperature at a high spatial and temporal resolution (Dehghannya et al. 2010), and it has been used to study the cooling process of strawberry (Ferrua and Singh 2009a, b, c; Nalbandi et al. 2016), grape (Delele et al. 2013a, b), citrus (Defraeye et al. 2013, 2014), kiwifruit (O’Sullivan et al. 2016), cauliflowers (Bideau et al. 2018)) and apple (Han et al. 2016, 2018).

Although there is no published official statistics, most producers in Iran are always complaining because they are suffering from the low benefits of mushroom production. The lack of suitable postharvest technologies for mushroom results in considerable losses, burdening high production expenses to the producer and reducing the outcome. Forced-air cooling could be regarded as a promising technology because firstly, compared with vacuum cooling, it has low investment cost. Secondly, it can be used for a wide variety of agricultural products which justifies its installation economically. A comprehensive knowledge of the process is the first step for efficient designing of the process. Therefore, this study is aimed to simulate the forced-convective cooling of mushroom so as to achieve a thorough understanding of the flow regime and heat transfer in the mushroom package. In fact, the main purpose of this study was to determine the main physical mechanisms controlling and affecting the process. Besides, the experiments were done to validate the model and verify its accuracy. It is expected that the current model allows researchers and engineers to investigate the influence of designing and operational factors such as package design, air velocity as well as air temperature and to design a high efficient process for mushroom cooling.

## Materials and methods

### Experiment

#### Sample preparation

Fresh mushrooms were bought from local market in Potsdam. Mushrooms of similar size with the average weight of 37.5 ± 1.5 g were chosen with minimum apparent variability. When choosing mushrooms, lack of head opening was considered. Before cooling experiments, the mushrooms were brought in the room temperature (18 °C) to climatize and achieve almost uniform initial temperature of the experiments.

#### Experimental setup

**Fig. 1:**
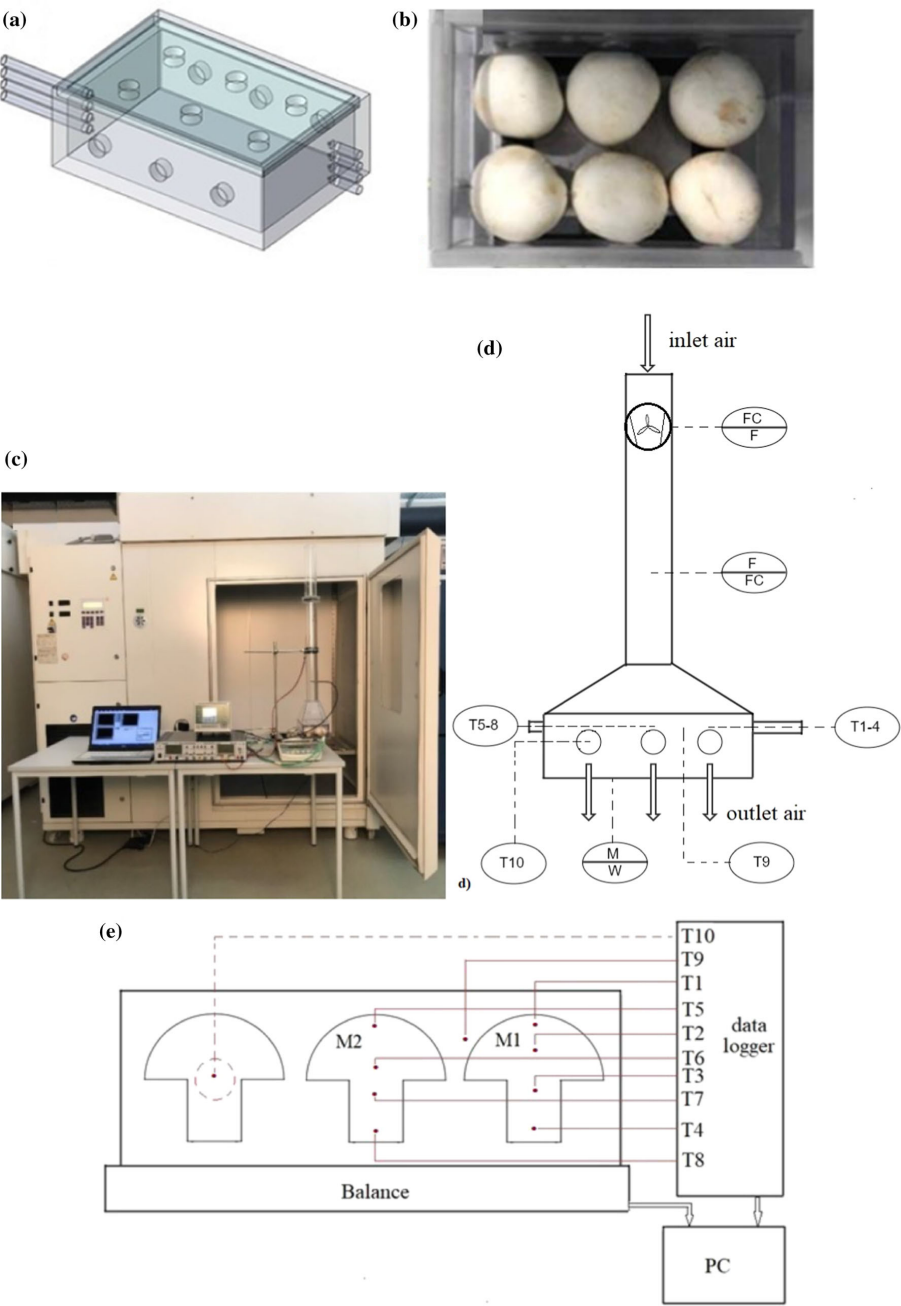
**a** Schematic of cooling box, **b** cooling box, **c** whole experimental set-up, **d** schematic of experimental set-up and **e** schematic of the cooling box which shows the position of the sensors

To validate the numerical model and evaluate its accuracy, a small laboratory scale cooling box was constructed from acrylic glass (Fig. 1a). The length, width, and height of the package were chosen to be of the value of 170, 115 and 55 mm, respectively, so that it could contain six mushrooms (Fig. 1b). To conduct cooling air into the box, six circular holes with a diameter of 15 mm were drilled into the top plate which could be opened and closed slidingly (Fig. 1a). Six circular holes of the same size were arranged in the lateral walls to allow the air to exit from the box, and so there was a continuous flow inside the box during the experiment. A vertical pipe followed by a funnel was designed and positioned at the top of the cooling box to provide and conduct a uniform air flow regime into the box (Fig. 1c). A centrifugal fan with adjustable voltage was used to generate and blow the desired airflow to the package (Fig. 1d). The air velocity in the vertical pipe was measured by a thermal anemometer with the accuracy of 0.04 m s^−1^ (FVAD 35 TH4, AHLBORN) which was positioned far enough from the fan so that the turbulence resulting from the rotating fan didn’t affect the velocity measurements. The juncture of package and funnel was completely sealed to ensure all the air would be directed to the package. The temperature data for mushrooms as well as the air were measured using thermocouples (type K, with the accuracy of 0.2 °C) for validation of the mathematical model. Inserting and fixing the thermocouples was the most challenging part of the experiments. For solving this problem, thin horizontal pipes were fixed at the cooling box to hold the thermocouples (Fig. 1a). The temperature and relative humidity of the cooling air were continuously monitored to ensure the stability of cooling conditions. The data acquisition was done by a data logger (ALMEMO 5690-2). The whole setup was stabilized by a stand and placed on the balance (KERN, PLJ4000-2M). Data acquisition was performed using the software LabView^®^.

#### Experimental plan

The experimental setup was maintained in cooling room at 2 °C for 2 h before the experiments to minimize its effect on the cooling process. Climatized samples were then put into the package and the measuring sensors were fixed as fast as possible. Due to the heterogeneous morphology of mushroom in head and stem, the average of four temperatures inside the mushroom was thought to be a good representative of mushroom temperature. Therefore, four thermocouples were inserted in such a way that they monitored the mushroom temperatures alongside its vertical axis (Fig. 1e). This temperature profile was measured at two mushrooms (one in the corner and one in the middle of the box). Two more thermocouples were used in order to measure the temperature of the air inside the box between mushrooms and at the outlet. After preparation, the experimental setup was then put on the balance in a cooling chamber (5 m × 4 m × 1 m) which was adjusted to a temperature of 2 °C to measure the mass continuously in order to quantify the effect of moisture loss as a function of time over the cooling period. By switching on the fan, airflow of 9 l min^−1^ was sucked from the cooling chamber and blown into the cooling box by the centrifugal fan. The cooling experiments were replicated for three times. All the measurements were recorded every 30 s. Since the velocity measurement inside the package is challenging, the model has been just validated by comparing the experimental temperature data against data from simulation.

### Mathematical modelling

In order to simulate the process, the 3D geometry of the cooling box (170 mm × 115 mm × 55 mm) including the six discrete mushrooms, was generated using ANSYS Design Modeler. For it, the following assumption was regarded: the shape of mushroom head and stem were considered to be hemispherical and cylindrical, respectively. Such simplifications in modelling are always needed in order to limit the grid number and hence computational costs considerably. It should be noted that this assumption was almost valid for the uniform mushrooms which had been selected for experimental study. Six circular holes with the diameter of 15 mm used as inlets were designed on top side of the box in such a way that the air entered the box exactly on top of head of each mushroom. The same holes positioned almost at half height of two reciprocal lateral sides were modeled as air outlets. The total open area for inlets and outlets was 5.42% and 3.38%, respectively.

Owing to the special design of the experimental setup, it could be assumed that the air flow distributed quite symmetrical both in the xy-plane and zy-plane. Therefore, the simulation model was reduced to one fourth of the box. This geometry was, then, meshed using ANSYS Meshing as an unstructured by a hybrid mesh involving both wedge and tetrahedral elements. The grid consisted of 259,024 cells and 88,441 nodes. Meshing the geometry seems to be one of the most important parts of each numerical simulation because in addition to the formulation of physical mechanisms governing the process, the accuracy of the numerical simulations depends largely on the mesh quality. To facilitate the meshing process and also to avoid the occurrence of negative volumes which prevents the solution running, consideration of a small gap between mushrooms and the walls of box was essential. Much finer mesh was used in regions where higher gradients were expected such as inlets and outlets. A boundary layer mesh was used in air-mushroom interfaces and on the box walls as well.

The simulation was performed in CFD commercial code Fluent 19.2, which is based on finite volume method, in two separate steps: In first step, the flow-field equations (continuity and standard Navier–Stokes equations) were solved using a steady solver, and in the next step, the energy transport was simulated in both solid phase and gas phase as a time dependent process. The obtained data from the first step were used as initial values for the latter and the flow field equations were switched off in transient mode.

#### Momentum equations of flow field

Dealing with agricultural product, many researchers regarded the complex geometry of packaging system as a porous medium (Zou et al. 2006a, b; Alvarez et al. 2003). However, this assumption cannot be justified anymore when the container-to-particle equivalent diameter ratio is less than 10. In this case, the transport phenomena can be largely affected by the local airflow pattern (Ferrua and Singh 2009a). In fact, assuming a porous medium results in some errors in the predicted values, and so the data are not reliable enough to be used for further improvement (Dehghannya et al. 2010; Ferrua and Singh 2009a). According to Ferrua and Singh (2009a), a transition occurs from laminar to turbulent flow when the airflow rate is approximately 20% higher than that is used in cooling industry in packages with the container-to-particle equivalent diameter ratio under 10. Therefore, by considering the air as a Newtonian fluid and a steady, laminar and incompressible flow, the following equations were solved for each discrete control volume:

1 $$\partial u_{i} /\partial x_{i} = 0,$$

2 $$u_{j} \left( {\partial u_{i} /\partial x_{i} } \right) = - \left( {1/\rho_{a} } \right)\left( {\partial P/\partial x_{i} } \right) + \upsilon_{a} \left( {\partial^{2} u_{i} /\partial x_{j} \partial x_{j} } \right),$$

where *u* and *P* stand for the velocity (m s^−1^) and pressure field (Pa), respectively. $\rho_{a}$ is the air density (kg m^−3^) and $\upsilon$ accounts for the kinematic viscosity (m^2^ s^−1^).

The flow rate commonly used in the cooling industry for fruits and vegetables is between 1 and 2 l s^−1^ per kilogram product (Defraeye et al. 2013). Accordingly, a constant air velocity of 0.15 m s^−1^ was imposed at the inlets, and the ambient atmospheric pressure was imposed at the outlets. The package walls and mushroom surfaces were modeled as no-slip walls with zero roughness, meaning that the fluid velocity relative to the boundary is zero.

#### Energy equation within the mushroom and cooling air

**Fig. 2:**
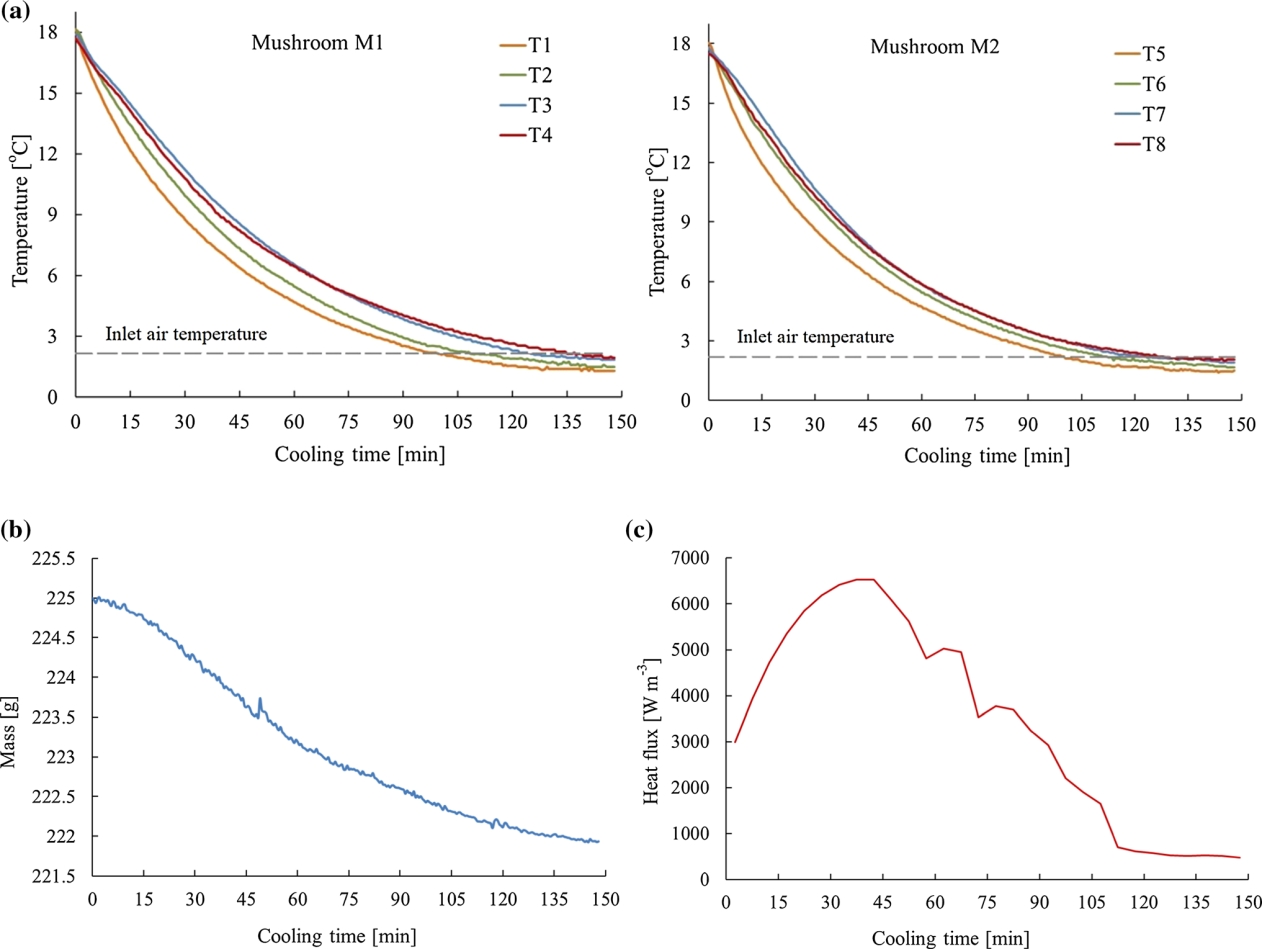
Experimental results of **a** temperature profile within the mushrooms M1 and M2, **b** total masses of mushrooms as a function of cooling time, and **c** average of specific volume heat flux over cooling time

Since the Biot number of most agricultural products lies between 0.1 and 10, they cannot be accounted for lumped system in which a uniform temperature distribution would be observed during a thermal process. Therefore, the energy equation (Eq. 3) was solved in order to achieve the temperature distribution within the mushrooms. Convective heat transfer and removal of latent heat are two simultaneous mechanisms which result in mushroom cooling during the forced-air cooling process. The former occurs due to the temperature gradient between the mushrooms and the bulk flow of the air, while the latter is associated with moisture loss from the mushroom surface. An ideal model, including all these mechanisms and their interactions, however, is not usually applicable due to the complexity of the flow field in the package and the amount of information contained in the model (Ferrua and Singh 2009a). Hence, to simplify the model and avoid an unsteady mass transfer simulation, the energy transported due to moisture loss is included by DEFINE_SOURCE macro in energy transport equation as a source term. To this, the total cooling time was subdivided into equal small time spans and the mass loss for each time span was extracted from experimental data (Fig. 2b). The latent heat due to the mass loss was then calculated based on the average surface temperature of mushrooms obtained from simulation for the respective time span (Fig. 2c) and added to the energy equation of mushroom (Eq. 3). As the rate of mass loss changed by the time, this source term was also changing as a function of cooling time. It was assumed that no shrinkage occurs as a result of water evaporation during the process. Radiation (Ferrua and Singh 2009a; Defraeye et al. 2013; Delele et al. 2013a, b) and respiration effects were not taken into account in the energy equation because they were negligible compared to convective heat transfer. The product respiration generally represents less than 1% of the total energy transferred during forced –air cooling application (Chuntranuluck et al. 1998). Based on these assumptions, the energy equation within the mushroom is represented as follows:

3 $$\left( {\rho C_{p} } \right)_{p} \left( {\partial T_{p} /\partial t} \right) = k_{p} \nabla^{2} T_{p} - Q,$$

where $\rho$ is the density (kg m^−3^). C_p_ and k stand for the specific heat capacity (J kg^−1^ K^−1^) and the thermal conductivity (W m^−1^ k^−1^), respectively. Subindex p is representative for the product. The density of mushroom as assumed to be constant with a value of 860 kg m^−3^ on average (McGarry and Burton 1994) during the process. Other properties, namely specific heat capacity and thermal conductivity, were varying as a function of temperature according to Eqs. 4 and  5, respectively (Shrivastava and Datta 1999):

4 $$C_{p} = 731.72 + 9.2 T,$$

5 $$k = 455.6 \times 10^{ - 3} + 2.348 \times 10^{ - 4} T,$$

where T is the volume-averaged temperature (K) of mushroom.

Theoretically, the energy transport within the fluid region takes place by convection, conduction as well as the energy transport resulting from diffusion of water vapor. However, because of the continuous airflow through the package, only a small amount of water is added to the air per unit of time (less than 0.03% of the total mass of air forced through the system) and so, enthalpy transferred which is accompanied by diffusion of water vapor is thought to be negligible (Hoang et al. 2003; Ferrua and Singh 2009a). Richardson number was obtained to be 0.23, as well, implying that the buoyancy effect can be neglected. Therefore, Eq. 6 represents the energy transport within the cooling air:

6 $$\left( {\rho C_{p} } \right)_{a} \left[ {\left( {\partial T_{a} /\partial t} \right) + \nabla \cdot uT_{a} } \right] = k_{a} \nabla^{2} T_{a} ,$$

where $\rho$ is density (kg m^−3^). C_p_ and k stand for specific heat capacity (J kg^−1^ K^−1^) and thermal conductivity (W M^−1^ k^−1^), respectively. Subindex a is representative for the air. The properties of the air were considered to be the same as those for dry air depending on the cooling temperature. Within the mushrooms and cooling air, the energy equations were then solved as conjugate heat transfer.

The inlet temperature of the air entering the box was equal to the temperature in the cooling chamber (2 °C) which was adjusted in the typical range of cooling facilities. The initial temperature of the mushrooms was patched to the same value of starting temperature of the mushroom in the experiment (18 °C). The box walls were considered as isolated walls, meaning that no heat loss occurred through the package walls. To the author’s knowledge, there is no commercially applied forced-air cooling system for mushroom. That is why the operating conditions of the process used for simulation were only based the literature on forced-air cooling of agricultural crops.

Mesh independency test was done by generating finer mesh continuously. This was done after solving the flow field. The test was conducted using velocity values measured alongside a vertical line passing through the center line of one exit vent which were compared for different mesh number 190,275, 259,024 and 324,821. Finally, the grid with the total number of 259,024 was distinguished to be fine enough not to affect the flow field.

## Results and discussion

### 1. Experimental results and model validation

The temperature data recorded for mushrooms M1 and M2 during experiments are presented in Fig. 2.a. The average initial masses of M1 and M2 were 37.95 and 37.35 g, respectively. The average diameter of the head was 52 and 50.1 mm for M1 and M2. M1 has a stem with the diameter 19.5 mm and the height 17.9 mm, and the values of M2 were 21.2 and 18.3 mm, respectively. As can be seen from the graphs, uniform temperature profiles were achieved with the value of 2 °C (cooling temperature) in the mushrooms after two and half hours. By comparing the obtained temperature of sensors over the time, the heterogeneous texture of mushroom can be clearly approved. Although the assumption of non-lumped system for mushroom has been justified, the cooling behavior of head and stem is quite different. The temperature pattern was relatively uniform in mushroom stem during the experiment, while big temperature difference (about 1 °C) was obtained for thermocouples positioned in head.

These results clearly confirm that data obtained from one thermocouple cannot be regarded as a good representative for the mean mushroom temperature. On the other hand, a point by point comparison was not applicable because both the heterogeneity of the mushroom morphology and the positioning of the thin thermocouples made it difficult to determine the accurate inserting point of sensors inside the mushroom. Hence, the average of each four temperatures was assumed to be a suitable criterion for comparison with the volume-averaged temperature obtained from simulation.

Figure 2b shows the average mass loss of the whole sample during the experiments. The mushrooms experienced 1.29% water loss which plays a key role in the cooling process. Salamat et al. (2020) reported the mass losses of 1.64–2.01% during forced-air cooling of mushroom when the cooling temperature changed from 2 to 10 °C at higher flow rates compared to this experiment. However, it is worth pointing out that within the air velocity range below 0.25 m s^−1^, mass loss is not affected by air velocity (unpublished results). The independency of mass loss on air flow rate has also been observed by Han et al. (2018) during forced-convective cooling of apples. As it can be seen in the diagram, the mass loss took place over the whole cooling period with a decreasing rate, suggesting that the associated heat flux was varied accordingly. Hence, the amount of latent heat of evaporation in each time span was estimated according to the mass loss and added to the energy equation of mushroom rather than applying a constant heat flux value over the cooling period (Fig. 2c). It should be noted that although the mass loss decreased over cooling time, it will continue if the surrounding air is not close to saturation. The mass loss has considerable impact on the saleable weight. On top of that, moisture losses of 4–5% would result in spongy texture and visible shriveling of the fruit (Aswaney 2007). Moisture loss during the forced-convective cooling is almost indispensable and its amount varies from little to amounts considerable enough to cause visible shriveling or wilting. However, providing the air with high relative humidity of 90–95% and avoiding unnecessary air flow after achieving the desired product temperature are of measures to be taken to prevent excessive moisture loss and shrinkage.

**Fig. 3:**
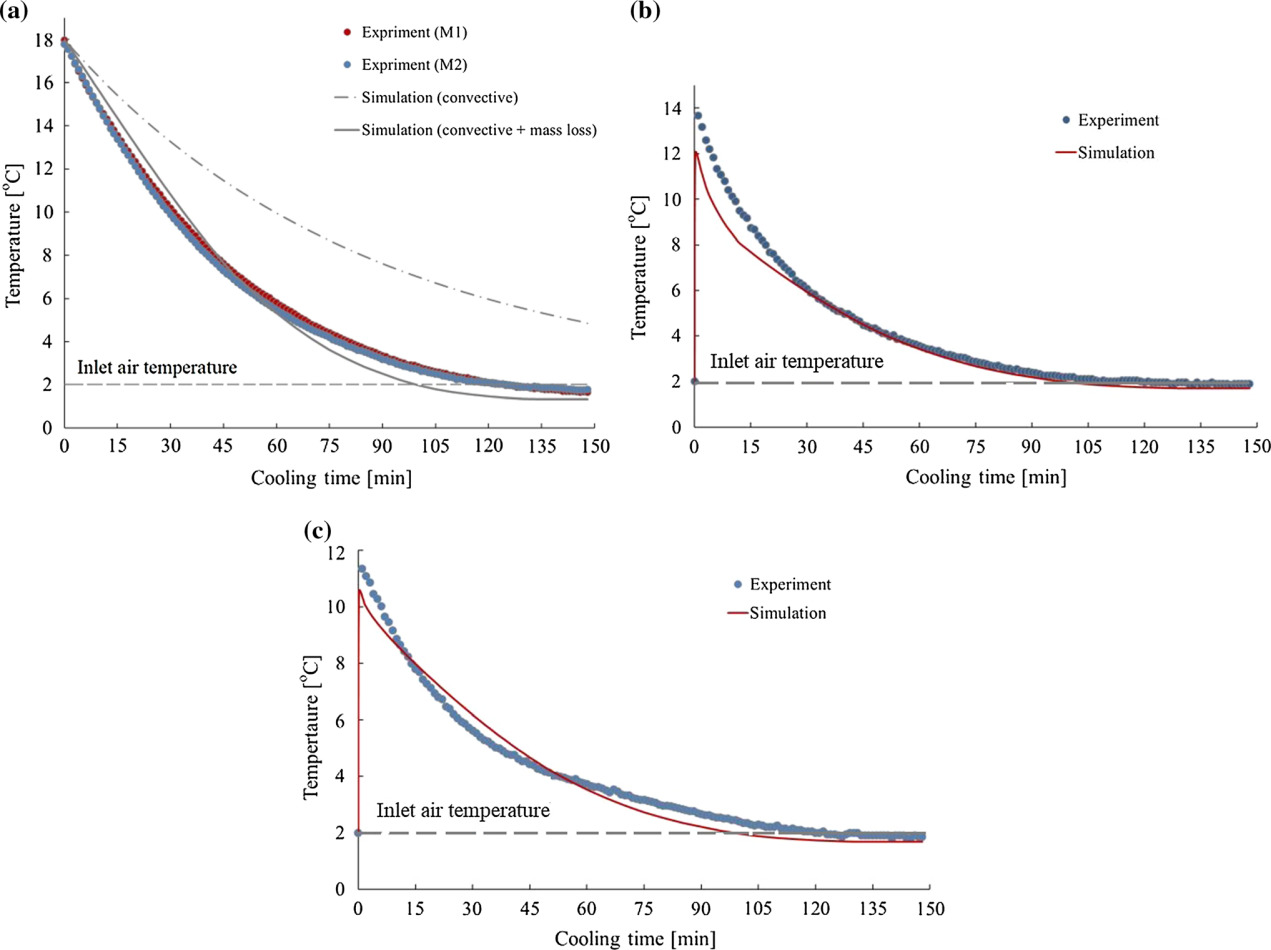
**a** Average temperature of mushroom over cooling time. Measuring values denote mean temperatures obtained from the four sensors in each mushroom. Simulated results indicate volume-averaged temperatures. **b** Air temperature in a certain point inside the box (T9) and **c** at the outlet (T10)

Figure 3a represents the experimental and simulation data, with and without latent heat, attained for the average temperature of mushroom. As it can be seen clearly, a good agreement between the experimental results and numerical data was achieved when the source term of latent heat is taken into account (RMSE was 3.8% and 3.9% for M1 and M2, respectively). Whereas, when ignoring the latent heat of evaporation, the mushrooms do not reach the cooling temperature even after two and half hours of cooling time whereas the process was completed in this point. Salamat et al. (2020) showed that the time needed for cooling the mushrooms at 2 °C was 1.7 h. The faster process could be because a higher air flow rate was used. In addition, an open top package used by them facilitated the interaction between air and mushrooms.

From 12.775 kJ heat exchanged during the cooling process, 7.5 kJ was devoted to water evaporation, and this contribution will increase for lower air velocities due to the independency of mass loss on magnitude of air velocity. It seems that the magnitude of this effect, also, depends strongly on the commodity and its capability of water loss. When modelling the forced-convective cooling of strawberry, latent heat of evaporation has been estimated through simultaneous solving of mass transfer within the fruits and added to the boundary conditions (Ferrua and Singh 2009a) while it has not been taken into account for citrus (Defraeye et al. 2013). Bideau et al. (2018) also reported that, besides convective heat transfer, evaporation and radiation occurred in cauliflower cooling and they couldn’t be neglected.

Figure 3b, c compare the results obtained from numerical model and experiments for air temperature inside the box (RMSE = 5.5%) and at the outlet (RMSE = 4.2%). Good agreement was obtained between the experimental data and numerical results. However, observed differences can be attributed to the errors in data measuring by thermocouples which occur because of the airflow effects. As thermocouples are very thin and long (about 100 mm), they were easily affected by air flow, especially by the higher velocities at the outlet.

The numerical model predicted very similar volume-averaged temperatures over cooling time for both mushrooms while small differences were observed between them in the experiments. These differences can be explained by positioning of sensors and variability of mushrooms. In addition, the same velocities were imposed in all inlets. However, it is likely that very small changes exist between velocity values. In order to remove the effect of product variability and reduce the complexity of experimental setup, product simulator can be used for validation. However, this is feasible provided that evaporation can be neglected which is not justified for some products like mushroom.

**Fig. 4:**
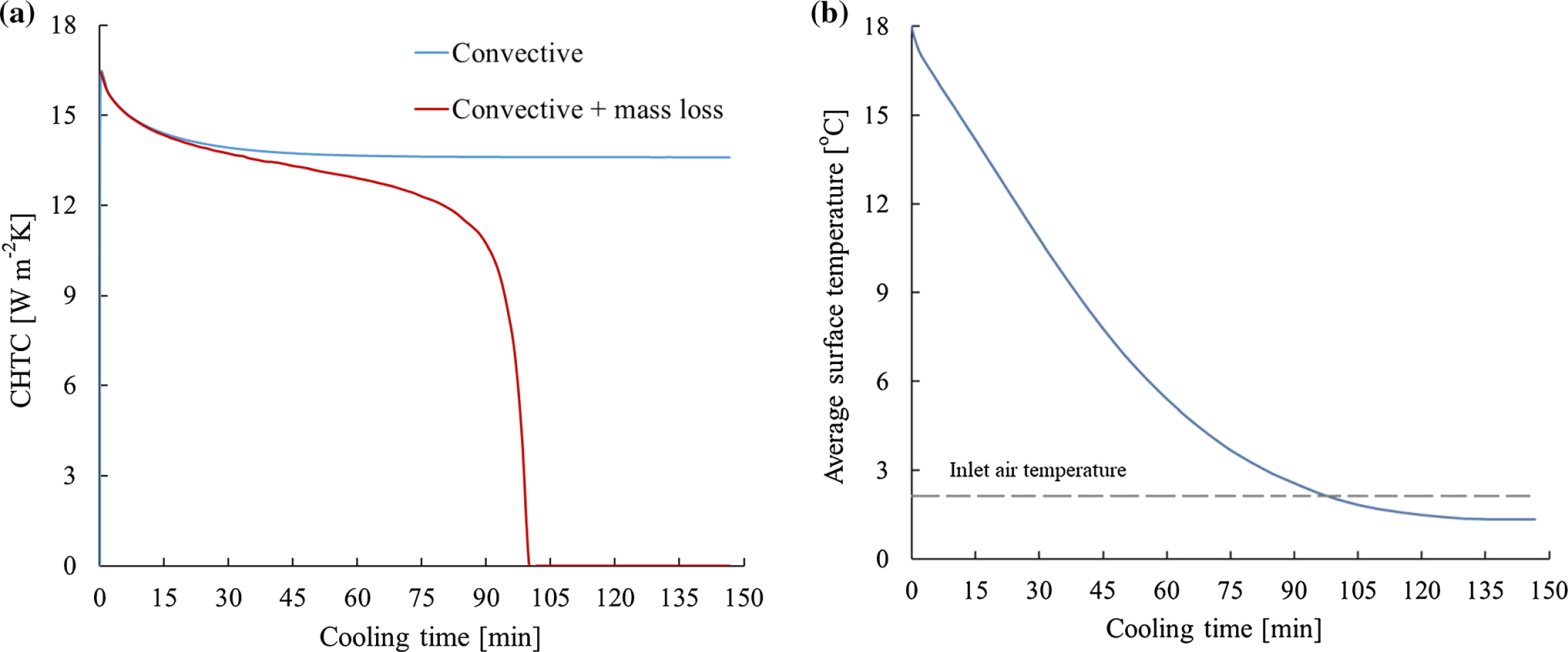
**a** Average convective heat transfer coefficient (CHTC), and **b** average surface temperature of mushroom

From the simulation results, the convective heat transfer coefficient (CHTC) has been calculated as an average value over the mushroom surfaces, the results of which are illustrated in Fig. 4a over the cooling time. In the diagram, both cases of convective cooling as well as convective and evaporative cooling are depicted. When the convective heat transfer is the only heat transfer mode, CHTC approaches a constant value after a short time, implying that the temperature boundary layer was fully developed. Whereas, adding the varying source term to the energy balance of the interface continuously affected boundary layer and caused different trend in CHTC. It continuously decreased during the first 90 min and then steeply declined to almost zero. This steep decrease can be attributed to the strong reduction of the average surface temperature of mushroom, which nearly approached the inlet air temperature (Fig. 4b). Thereby, the temperature gradient of convective heat transfer drastically reduced resulting in a very low CHTC value.

Amara et al. (2004) experimentally investigated the cooling process of spherical products at low air velocities and reported that CHTC was significantly affected by the position of the products inside the package. For spheres placed in rows close to the inlet and outlet, the CHTC value would be changing owing to the effects of reduction and expansion of air passage, whereas it was almost constant in the centre part of the package where the airflow was fully developed. They also stated that although temperature difference had not considerable effect on CHTC, the velocity increment resulted in an increased CHTC. It is worth mentioning that, however, the relationship between air velocity and heat transfer is nonlinear (Amara et al. 2004; Han et al. 2016). Therefore, velocity increasing is recommended to a threshold which needs to be accurately investigated for each cooling conditions.

### Simulation results

**Fig. 5:**
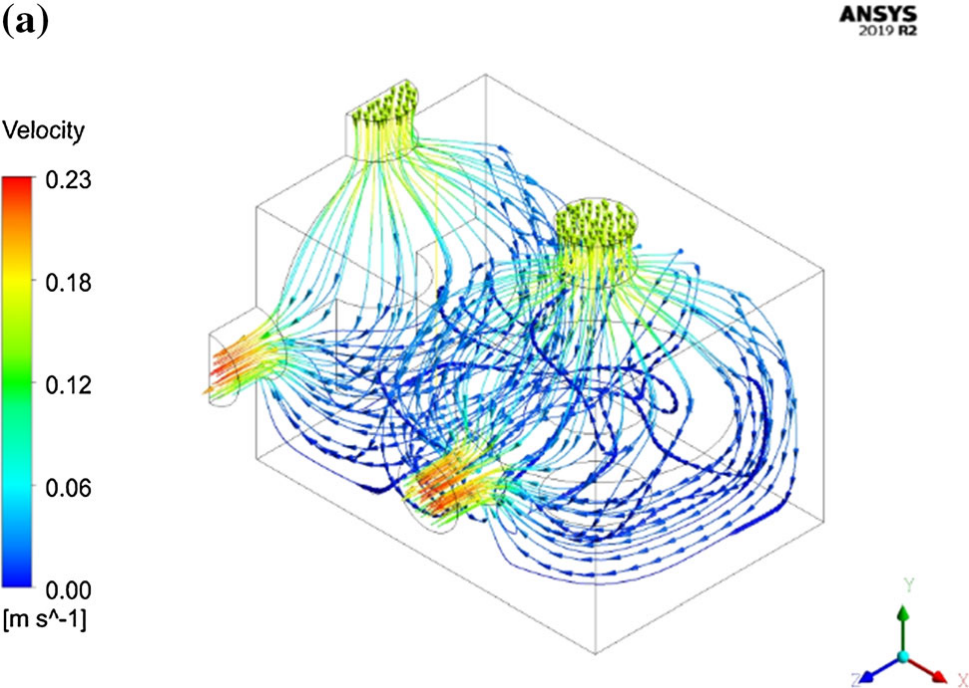

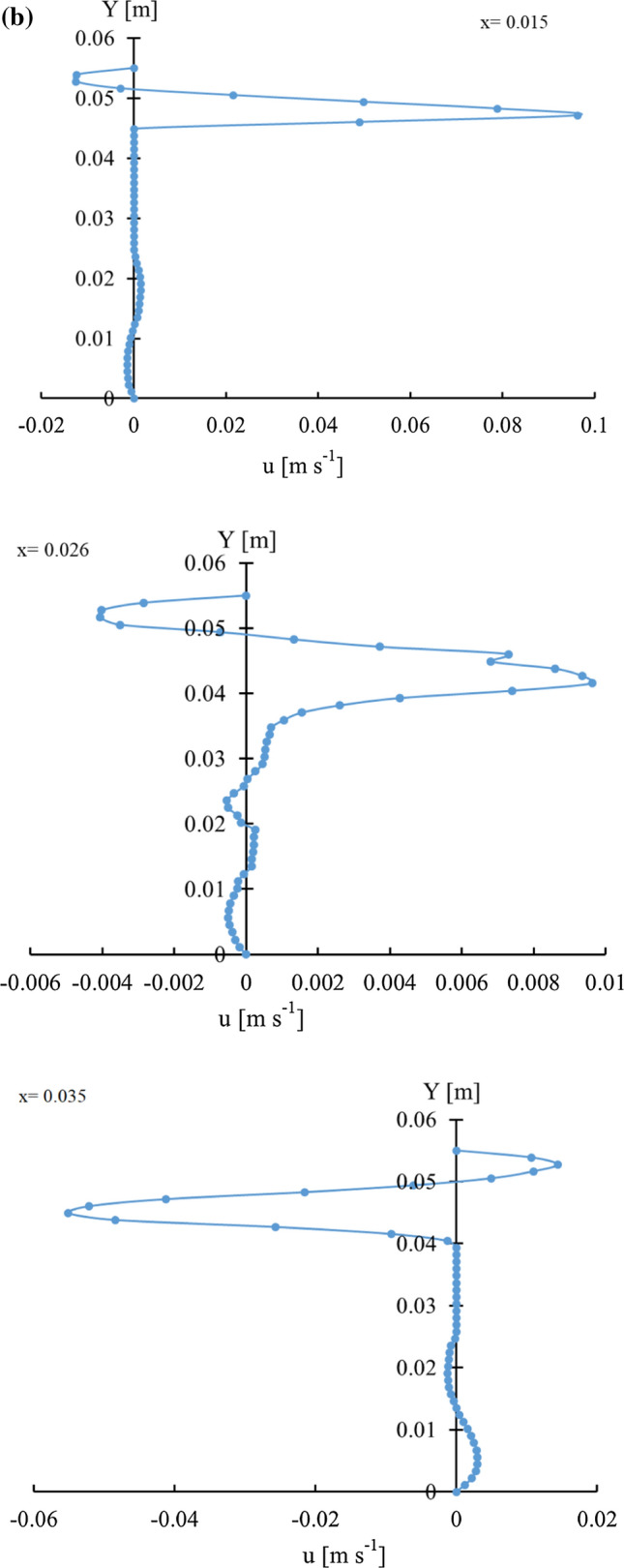

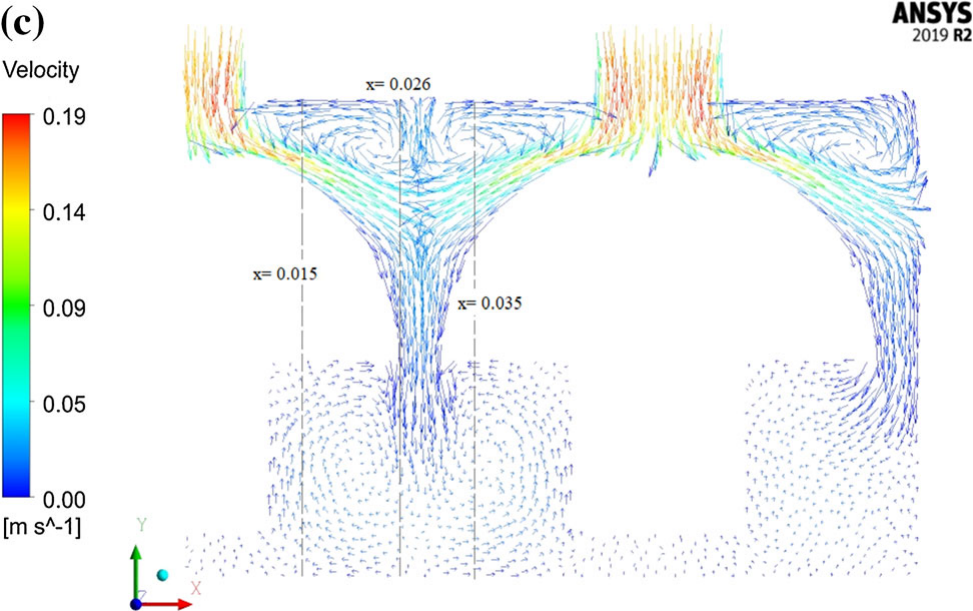
**a** The velocity vectors inside the package, **b** velocity magnitude alongside 3 vertical line in the package and **c** velocity vectors in a longitudinal section (in x–y plane)

As it was mentioned before, CFD provides some fundamental information about the air flow behavior and heat transfer processes provided that the developed model can be accurately validated. Such information as it is well documented in the literature is very useful and essential for design and optimization of the cooling process. The current numerical model predicted a quite nonhomogeneous air flow velocity field over the computational domain. Figure 5a represents the velocity vectors inside the cooling box. As expected, the highest air velocities were predicted to take place at the inlets and outlets with magnitudes of 0.18 and 0.23 m s^−1^, respectively. The velocity of the air in the upper part of the box was quite high compared to that in the lower half. The numerical model was well capable of detecting a dead region at the bottom part of the box in which the air seems to be almost stagnant. In Fig. 5b which shows the velocity magnitude along three lines parallel to the vertical axis of mushroom, this zone can be easily distinguished by low velocity values. In addition, a low velocity circular airflow was detected in the vicinity of mushroom stem which can result in air blocking in these regions. Circular movement of the air as well as dead zones were created as a result of combined effect of airflow system and mushroom geometry. Positioning inlets at the top of each head with this special geometry, especially where the distance between mushrooms are minimum, causes two inlet air streams moving along the y-axis. After the air streams reach the lower part of the package, the mushroom stems behave like baffles, therefore a circular flow is created (Fig. 5c). Existence of such regions in addition to negative effect on cooling uniformity in general, will be of big disadvantage especially for products with high moisture loss because the accumulation of water vapor may lead to water condensation. The presence of water is more likely to be a main reason of perishability in some fruits and vegetables because it provides suitable growth conditions for some fungi. It should be noted that this package design (Fig. 1a) was especially developed for experimental studies with the necessity to install thermocouples for measuring temperature distributions. However, it is not recommended for practice as it didn’t provide suitable airflow. The current model, in turn, gives designers the possibility to easily investigate and develop the efficient cooling equipment.

**Fig. 6:**
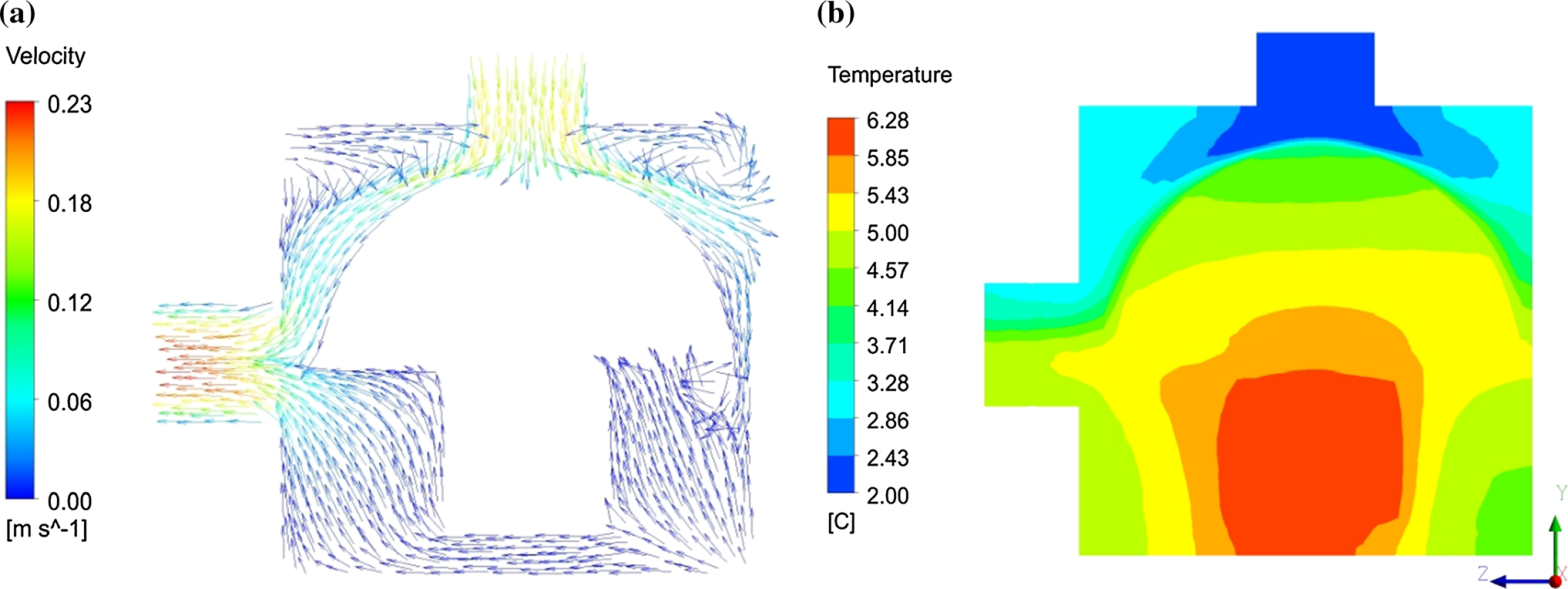
**a** The velocity vectors and **b** the temperature contours for intersectional plane (in y–z plane) inside the package after 1 h of cooling time

By comparing Fig. 6a and b, which respectively represent the velocity vectors and the temperature contour over a cross section after one hour of cooling time, the effect of local airflow behavior on cooling process can be easily understood. The mushroom head cooled faster than the stem because it was subjected to air with higher velocity and lower temperature. Uneven airflow regime inside the horticultural packages and its impact on temperature pattern during convective-air cooling has been repeatedly reported in the literature. The results imply that CFD modelling provides useful information about airflow behavior that is significantly affected by package structure and vent designs. Ferrua and Singh (2009a) identified a preferential pathway inside the clamshells, occurring due to coincidence of inlet and outlet vents with its headspace, which leads to 46 ± 5% of the air entered the clamshells bypassed the commodities. This effect is also visible in Fig. 6b illustrating the temperature profile, which shows a low temperature region in the upper part of the outlet. Defraeye et al. (2013) also reported such pathways when simulating the cooling process of citrus. The occurrence of such low flow resistance pathways can be easily detected using CFD compared to expensive, time-consuming experiments and must be avoided because it brings about nonhomogeneous cooling which is not acceptable at all. In addition, it reduces energy efficiency as it prevents all heat transfer potential of the air being used since the airflow rate through the fruit decreases and as a result, convective heat transfer resistance increases. Well-distributed vents on the package are an important prerequisite to achieve a homogeneous cooling (Zou et al. 2006b; Han et al. 2015; Dehghannya et al. 2011).

According to the Han et al. (2015), there was a temperature difference of 8 °C between two layers of apples in the package. This may also cause overcooling and chilling injury of the products subjected to higher airflow while the other products are not cooled enough. In addition to its influence on cooling efficiency in terms of cooling rate, vents design has a significant effect on total pressure drop and so energy consumption of the system. Increasing vent area not only enhances both cooling rate and cooling uniformity, but also reduces energy consumption through decreased pressure drop (Delele et al. 2013a, b). Nevertheless, mechanical strength of package is affected to large extent by the area of vents. An efficient ventilated package should provide a fast uniform cooling and protect the products against mechanical injury at the same time.

## Conclusion

A mathematical model was developed in order to determine the prevailing heat transfer modes among all physical mechanisms that occur during the forced-convective cooling of mushrooms, and it was strictly validated by experimental data. Both, water evaporation and convective heat transfer resulting from temperature gradients between cooling air and mushroom were found to be determinant in mushroom cooling. The results showed that the model provided a fundamental understanding of the complex transport phenomena within the individual package. Hence, the model has the utilization potential to optimize such a process in terms of package design, cooling air conditions and air flow system. Investigation of the effects of these factors using numerical model may meet the limitations of doing expensive, time-consuming experiments, especially on agricultural products with an extensive variability.

## List of symbols

$C_{p}$: Specific heat capacity (J kg^−^^1^ K^−^^1^)
$$k$$: Thermal conductivity (W m^−^^1^ K^−^^1^)
$$P$$: Pressure (pa)
$Q$: Heat source term (W m^−^^3^)
$$t$$: Time (s)
$$T$$: Temperature (°K)
$$u$$: Velocity (m s^−^^1^)
$x_{i}$: Cartesian tensor notation of the spatial coordinates
$$\rho$$: Density (kg m^−^^3^)
$$\upsilon$$: Kinematic viscosity (m^2^ s^−^^1^)

## Subindexes

$$a$$: Air
$$p$$: Product

## References

[CR1] Alvarez G, Bournet PE, Flick D (2003). Two-dimensional simulation of turbulent flow and transfer through stacked spheres. Int J Heat Mass Transf.

[CR2] Amara SB, Laguerre O, Flick D (2004). Experimental st 398 udy of convective heat transfer during cooling with low air velocity in a stack of objects. Int J Therm Sci.

[CR3] Aswaney M (2007) Forced—air precooling of fruits and vegetables. Air Cond Refrig J 57–62

[CR4] Bideau PL, Noel H, Yassine H, Glouannec P (2018). Experimental and numerical studies for the air cooling of fresh cauliflowers. J Appl Therm Eng.

[CR5] Burton KS, Frost CE, Atkey PT (1987). Effect of vacuum cooling on mushroom browning. Int J Food Sci Technol.

[CR6] Chuntranuluck S, Wells CM, Cleland AC (1998). Prediction of chilling times of foods in situations where evaporative cooling is significant. Part 1. Method development. J Food Eng.

[CR7] Defraeye T, Lambrecht R, Tsige AA, Delele MA, Opara UL, Cronjé P, Verboven P, Nicolai B (2013). Forced-convective cooling of citrus fruit: package design. J Food Eng.

[CR8] Defraeye T, Lambrecht R, Delele MA, Tsige AA, Opara UL, Cronjé P, Verboven P, Nicolai B (2014). Forced-convective cooling of citrus fruit: cooling conditions and energy consumption in relation to package design. J Food Eng.

[CR9] Dehghannya J, Ngadi M, Vigneault C (2010). Mathematical modeling procedures for airflow, heat and mass transfer during forced convection cooling of produce: a review. Food Eng Rev.

[CR10] Dehghannya J, Ngadi M, Vigneault C (2011). Mathematical modeling of airflow and heat transfer during forced convection cooling of produce considering various package vent areas. J Food Control.

[CR12] Delele MA, Ngcobo MEK, Getahun ST, Chen L, Mellmann J, Opara UL (2013). Studying airflow and heat transfer characteristic of a horticultural produce packaging system using a 3-D CFD mode. Part I: model development and validation. J Postharvest Biol Technol.

[CR13] Delele MA, Ngcobo MEK, Getahun ST, Chen L, Mellmann J, Opara UL (2013). Studying airflow and heat transfer characteristic of a horticultural produce packaging system using a 3-D CFD mode. Part II: effect of package design. J Postharvest Biol Technol.

[CR14] Ferrua MJ, Singh RP (2009). Modelling the forced-air cooling process of fresh strawberry packages. Part I: numerical model. Int J Refrig.

[CR15] Ferrua MJ, Singh RP (2009). Modelling the forced-air cooling process of fresh strawberry packages. Part II: experimental validation of the flow model. Int J Refrig.

[CR16] Ferrua MJ, Singh RP (2009). Design guidelines for the forced-air cooling process of strawberries. Int J Refrig.

[CR17] Han JW, Zhao CJ, Yang XT, Qian JP, Fan BL (2015). Computational modeling of airflow and heat transfer in a vented box during cooling: optimal package design. J Appl Therm Eng.

[CR18] Han JW, Badia-Melis R, Yang XT, Ruiz-Garcia L, Qian JP, Zhao CJ (2016). CFD simulation of airflow and heat transfer during forced-air precooling of apples. J Food Process Eng.

[CR19] Han JW, Zhao CJ, Qian JP, Ruiz-Garcia L, Zhang X (2018). Numerical modelling of forced-air cooling of palletized apples: integral evaluation of cooling efficiency. J Food Process Eng.

[CR20] Hoang ML, Verboven P, Baelmans M, Nicolaï BM (2003). A continuum model for airflow, heat and mass transfer in bulk of chicory roots. Trans ASAE.

[CR21] Iqbal T, Rodrigues FA, Mahajan PV, Kerry JP (2009). Effect of time, temperature, and slicing on respiration rate of mushrooms. J Food Sci.

[CR22] Kader AA (2002). Postharvest technology of horticultural crops, 3rd edition.

[CR23] Lagnika C, Zhang M, Nsor-Atindana J, Bashari M (2012). Effects of ultrasound and chemical treatments on white mushroom (*Agaricus bisporus*) prior to modified atmosphere packaging in extending shelf-life. J Food Sci Technol.

[CR24] McGarry A, Burton KS (1994). Mechanical properties of the mushroom, *Agaricus bisporus*. J Mycol Res.

[CR25] Nalbandi H, Seiiedlou S, Ghasemzadeh HR, Ranjbar F (2016). Innovative parallel airflow system for forced-air cooling of strawberries. J Food Bioproducts Process.

[CR26] O’Sullivan J, Ferrua MJ, Love R, Verboven P, Nicolai B, East A (2016). Modelling the forced air cooling mechanisms and performance of polylined horticultural produce. J Postharvest Biol Technol.

[CR27] Pathare PB, Opara UL, Vigneault C, Delele MA, Al-Said FAJ (2012). Design of packaging vents for cooling fresh horticultural produce. J Food Bioprocess Technol.

[CR28] Salamat R, Ghassemzadeh HR, Ranjbar F, Jalali A, Mahajan P, Herppich W, Mellmann J (2020). The effect of additional packaging barrier, air moment and cooling rate on quality parameters of button mushroom (*Agaricus bisporus*). Food Packag Shelf Life.

[CR29] Shrivastava M, Datta AK (1999). Determination of specific heat and thermal conductivity of mushrooms (*Pleurotus florida*). J Food Eng.

[CR30] Tao F, Zhang M, Hangqing Y, Jincai S (2006). Effects of different storage conditions on chemical and physical properties of white mushrooms after vacuum cooling. J Food Eng.

[CR31] Thompson J, Cantwell M, Arpaia ML, Kader A, Crisosto C, Smilanick J (2001). Effect of cooling delays on fruits and vegetables quality. Perish Handl Q.

[CR32] Wu W, Häller P, Cronje P, Defraeye T (2018). Full-scale experiments in forced-air precoolers for citrus fruit: impact of packaging design and fruit size on cooling rate and heterogeneity. J Biosyst Eng.

[CR33] Xiangyou W, Jincui T, Juan W (2014). Effect of recooling temperature on physiological quality of cold stored *Agaricus bisporus*. Int J Agric Biol Eng.

[CR34] Zhao CJ, Han JW, Yang XT, Qian JP, Fan BL (2016). A review of computational fluid dynamics for forced-air cooling process. J Appl Energy.

[CR35] Zou Q, Linus UO, McKibbin RA (2006). CFD modelling system for airflow and heat transfer in ventilated packaging for fresh foods: I. Initial analysis and development of mathematical models. J Food Eng.

[CR36] Zou Q, Linus UO, McKibbin RA (2006). CFD modelling system for airflow and heat transfer in ventilated packaging for fresh foods: II. Computational solution, software development, and model testing. J Food Eng.

