# Correction to: Dominant physical mechanisms governing the forced-convective cooling process of white mushrooms (*Agaricus bisporus*)

**DOI:** 10.1007/s13197-021-05166-6

**Published:** 2021-06-07

**Authors:** Razieh Salamat, Hamid Reza Ghassemzadeh, Seyed Faramarz Ranjbar, Jochen Mellmann, Hossein Behfar

**Affiliations:** 1 Department of Biosystem, University of Tabriz, Tabriz, Iran; 2 Department of Postharvest Technology, Leibniz Institute for Agricultural Engineering and Bioeconomy (ATB), Potsdam, Germany; 3 Department of Mechanical Engineering, University of Tabriz, Tabriz, Iran

## Correction to: J Food Sci Technol (October 2020) 57(10):3696–3707 https://doi.org/10.1007/s13197-020-04402-9

The article “Dominant physical mechanisms governing the forced-convective cooling process of white mushrooms (*Agaricus bisporus*)”, written by [Razieh Salamat, Hamid Reza Ghassemzadeh, Seyed Faramarz Ranjbar, Jochen Mellmann, Hossein Behfar, was originally published Online First without Open Access. After publication in volume 57, issue 10, page 3696–3707 the author decided to opt for Open Choice and to make the article an Open Access publication. Therefore, the copyright of the article has been changed to © The Author(s)Team’s 2020 and the article is forthwith distributed under the terms of the Creative Commons Attribution 4.0 International License, which permits use, sharing, adaptation, distribution and reproduction in any medium or format, as long as you give appropriate credit to the original author(s) and the source, provide a link to the Creative Commons licence, and indicate if changes were made. The images or other third party material in this article are included in the article’s Creative Commons licence, unless indicated otherwise in a credit line to the material. If material is not included in the article’s Creative Commons licence and your intended use is not permitted by statutory regulation or exceeds the permitted use, you will need to obtain permission directly from the copyright holder. To view a copy of this licence, visit http://creativecommons.org/licenses/by/4.0/.

The original article has been updated.

